# Unreliable Gut Feelings Can Lead to Correct Decisions: The Somatic Marker Hypothesis in Non-Linear Decision Chains

**DOI:** 10.3389/fpsyg.2012.00384

**Published:** 2012-10-09

**Authors:** Manuel G. Bedia, Ezequiel Di Paolo

**Affiliations:** ^1^Department of Computer Science, University of ZaragozaZaragoza, Spain; ^2^Ikerbasque – Basque Science FoundationBilbao Bizkaia, Spain; ^3^Centre for Computational Neuroscience and Robotics, University of SussexEast Sussex, UK

**Keywords:** dual system decision-making, affect, decision chains, dynamic decision-making, somatic marker hypothesis, discrete-time Markov chains

## Abstract

Dual-process approaches of decision-making examine the interaction between affective/intuitive and deliberative processes underlying value judgment. From this perspective, decisions are supported by a combination of relatively explicit capabilities for abstract reasoning and relatively implicit evolved domain-general as well as learned domain-specific affective responses. One such approach, the somatic markers hypothesis (SMH), expresses these implicit processes as a system of evolved primary emotions supplemented by associations between affect and experience that accrue over lifetime, or somatic markers. In this view, somatic markers are useful only if their local capability to predict the value of an action is above a baseline equal to the predictive capability of the combined rational and primary emotional subsystems. We argue that decision-making has often been conceived of as a linear process: the effect of decision sequences is additive, local utility is cumulative, and there is no strong environmental feedback. This widespread assumption can have consequences for answering questions regarding the relative weight between the systems and their interaction within a cognitive architecture. We introduce a mathematical formalization of the SMH and study it in situations of dynamic, non-linear decision chains using a discrete-time stochastic model. We find, contrary to expectations, that decision-making events can interact non-additively with the environment in apparently paradoxical ways. We find that in non-lethal situations, primary emotions are represented globally over and above their local weight, showing a tendency for overcautiousness in situated decision chains. We also show that because they tend to counteract this trend, poorly attuned somatic markers that by themselves do not locally enhance decision-making, can still produce an overall positive effect. This result has developmental and evolutionary implications since, by promoting exploratory behavior, somatic markers would seem to be beneficial even at early stages when experiential attunement is poor. Although the model is formulated in terms of the SMH, the implications apply to dual systems theories in general since it makes minimal assumptions about the nature of the processes involved.

## 1 Introduction

Psychological and neurobiological evidence accumulated over the last two decades has supported a dual system account of decision-making (Damasio, 1994; Epstein, 1994; Sloman, 1996; Bechara et al., 1997; Lieberman, 2000; Evans, 2003, 2008; Bechara and Damasio, 2005; Ferreira et al., 2006; Weber and Johnson, 2009; Morewedge and Kahneman, 2010). Under a wide range of circumstances the quality of decisions is enhanced by intuitive and affective processes that regulate and advantageously bias fast and automatic judgments. At the same time, investing effort on rational cost-benefit analysis beyond a certain limit does not accrue quality increments to the outcome of a decision either objectively or subjectively (e.g., Wilson and Schooler, 1991). On the contrary, distraction from thinking too much about pros and cons often has positive benefits for judgment quality (Betsch et al., 2001; Dijksterhuis et al., 2006; Usher et al., 2011).

In this paper we address the question of what determines the functional balance of between these processes and whether it is sufficient for answering this question to adopt a localist perspective on decision events or whether, on the contrary, a dynamical approach is required involving potentially non-linear interactions between cognitive and affective processes, and decision sequences.

Dual system theories postulate the existence of two distinct cognitive systems at play during decision-making (Evans, 2003, 2008): System 1, which is implicit, intuitive, affectively loaded, functionally faster, automatic, more concrete and situation-dependent, harder to report, and evolutionarily older although not necessarily independent of experience and System 2, which is deliberative, functionally slower, mostly sequential, involving conscious, conceptual, rule-based and/or linguistic processes, more domain-general, experience-dependent, easier to articulate, and evolutionarily more recent (most clearly evidenced in human reasoning capabilities).

The implicit processes in System 1 can in turn be further unpacked into relatively autonomous subsystems (Gore and Sadler-Smith, 2011). Some of these subsystems are supposed to be evolutionarily older, mostly experience-independent, associated with basic biological and reproductive needs and social constraints that often require rapid response. They tend to be relatively conservative, being rapidly deployed in situations perceived as risky and involving values such as self- and kin-protection and survival. Other subsystems rely more strongly on domain-specific learning and the degree of accumulated expertise. They often work by associating situations and affects and by incorporating the results of past decision-making events into embodied know-how. This know-how may at one point have been assisted by rational deliberation and rule-following but has now been incorporated into readily available habits and intuitions (Dreyfus, 2002). For this reason, experience-dependent affective processes can be better attuned to the actual risks of a situation and tend to produce less conservative, more fine-grained responses.

The somatic markers hypothesis (SMH; Damasio, 1994; Bechara and Damasio, 2005) offers a systems-level, neuro-anatomical account of the affective processes involved in this distinction. The SMH distinguishes between primary emotions corresponding to the first subsystem and secondary emotions corresponding to the second. The latter are based on experience-dependent patterns of bio-regulatory and visceral signals that act as bodily “markers” to produce a rapid indication of the valence and intensity of a situation based on past experiences.

Although a significant amount of theorizing has been dedicated to clarifying the functional relations between these systems, certain key issues have remained under-studied. For instance, how should we understand the relation between these systems at a functional level, especially in the context of a history of decision events or in the context of development? It seems that an optimal cognitive architecture would involve just the right context-dependent balance of primary emotions to conserve basic aspects of survival together with domain-specific, know-how related secondary emotions to act efficiently and avoid excessive cognitive load, all in combination with System 2’s deliberative, conceptual processes to deal with complex or novel problem-solving. But how is this balance to be determined? In this paper we claim and show by means of a non-linear Markov chain model that this question is highly dependent on whether we take a static vs. a dynamic view of decision-making, leading to radically different answers.

Everyday decision-making sometimes involves chains of decisions necessitating different local judgments and actions aimed at a global desired outcome. Real-world scenarios can be uncertain about the relation between local and global utility. Often an optimal route to a goal can be ridden with unforeseen problems or novel options that might be locally neutral or even detrimental but still lead faster to a good global solution. Moreover, the quality and value of options at the local level can interact non-trivially with decision-making processes themselves. This is the case, for instance in situations involving competition between different agents, or resource allocation or exploitation in time-varying circumstances, or interventions over time (like the case of a doctor prescribing a long-term treatment to bring a patient back to heath), or in cases of decisions that bias trajectories toward certain regions of the problem space that reinforce the use of the same decision strategies preventing further progression.

In a static, linear view, the question of the functional balance between the different processes would be answered by assuming that the optimal conditions for a single decision event (given its context) are applicable to groups of interlinked decision events. This is then resolved as the question of the appropriate adaptation of context-sensitivity of all the mechanisms as they are evaluated within a point situation. Such locally optimal decision-making has been described using various formal models (e.g., Bogacz et al., 2006; Bogacz, 2007). Extending this analysis to decision sequences is equivalent to treating decision events as semi-independent, thus assuming an additive, linear approach. At each point, a judgment will be influenced by previous decisions at most in that the current state depends on them or as a result of learning about the problem space. For instance, a series of negative outcomes might be followed by an increase in the exercise of caution as part of a general sampling and attunement to the statistics of the situation. But once this learning is achieved, a stationary situation is assumed to ensue: decisions are informed by the learned statistics and the quality of the problem space remains de-coupled from decision events. This introduces a de-coupling between the decision-making agent and the problem space as decision-making processes function based on perceptual inputs assumed independent of the very same processes.

However, it is possible for complex interactions to take place between events in a decision chain in ecologically relevant situations. It is also possible for the global utility not to be reflected necessarily in the maximization of local utility, but that several locally neutral (or locally negative) paths can lead to the desired end state. The combination of these two possibilities (non-additive interactions between decision events and ecological embeddedness) calls for a dynamical examination of decision chains.

A dynamical perspective brings new considerations besides local optimality to the question of the balance between the different systems. For instance, conservative System 1 processes may interact non-linearly with sequences of decision events resulting in less overall exploratory behavior, thus influencing negatively the timely development of well-attuned somatic markers. Conversely, even mal-adjusted secondary emotions may have a positive effect by breaking deadlocks caused by the amplification of cautionary decisions once we consider them in the context of several decision events. This in turn has consequences for understanding the evolution of secondary subsystems such as somatic markers. Presumably their initial mal-adjustment due to lack of experience would imply negative early effects from a static point of view: they would place the agent under unnecessary risk before it has a chance to improve its context-sensitivity with experience. Having to overcome such a potentially lethal developmental “valley” begs the question of how could somatic markers be favored during evolution in the first place.

We investigate these questions (the balance between processes and their positive or negative effects in a dynamical context) by proposing a non-linear model of decision-making using discrete-time Markov chains and expressed in terms of the Somatic Markers Hypothesis (SMH). The model, however, is applicable to dual systems theories in general. Its main components are the interactions between deliberative (rational, general, and hypothesis-driven) mechanisms, and two types of emotional mechanisms, one primary, linked to readily available, conservative emotional responses (originating in evolved adaptations) and one secondary involving domain-specific associations learned by experience. These are modeled using minimal assumptions in terms of their probabilistic effects, so that the main results are independent of the specific implementation of these systems, e.g., at the neuro-visceral level.

Contrary to what would be expected by the assumptions of linearity and de-coupling, our model shows that decision-making processes and environmental dynamics interact in apparently paradoxical ways. In particular, poorly attuned secondary emotions that by themselves would not locally enhance decision-making, can still produce an overall positive effect, dissolving in this way the evolutionary worries about the developmental valley. Somatic markers turn out to be beneficial even at early stages where experiential attunement is poor. Other findings confirm the amplification of cautionary effects of primary emotions and the decreasing marginal gain of investing in improving deliberative processes.

## 2 Modeling Decision-Making Processes

The modeling of decision-making processes is divided between the normative approach that seeks to establish how decisions should be made through the maximization of some utility (e.g., von Neumann and Morgenstern, 1947; Savage, 1954) and the descriptive approach that analyzes how decisions are actually made (Tversky, 1972; Kahneman and Tversky, 2000). The latter studies when and why decision-makers systematically violate principles of optimal decision-making (Rieskamp et al., 2006). This approach often relies on the following assumptions (Ratcliff and Smith, 2004): (i) a decision is expressed as a choice between two alternatives, so that the evidence in favor of one counts against the other, and (ii) the process involves “random sequential sampling” (Ashby, 1983; Ratcliff and Smith, 2004), i.e., the decision-maker receives stochastic successive samples in a sequential manner until a criterion of evidence is met. The optimal strategy for solving these data-accumulating models is inspired by “drift-diffusion” models in physics (Milosavljevic et al., 2010) where: (1) a “drift” process is caused by available evidence and (2) a “diffusion” process is caused by noise, so that (3) decisions are made when the relative evidence for one of the alternatives exceeds a pre-specified threshold (see Bogacz et al., 2006 for a review). In general, all the models of cumulative processes with stochastic properties are expressed using Markov chains (Smith, 2000), i.e., systems in which the current state is completely defined by the preceding one. A decision-maker calculates the expected utility of a possible decision for the state *t* + 1 as the sum of the probability of each possible outcome multiplied by the utility of each outcome at the state *t*.

For example, in a “single system” case, an agent is presented with two choices {*A*, *B*} with two possible outcomes each {*a*_1_, *a*_2_ ∈ *A*}, {*b*_1_, *b*_2_ ∈ *B*}, that occur with probabilities {*p_a_*_1_, *p_a_*_2_} and with {*p_b_*_1_, *p_b_*_2_} respectively.

In practice, the agent’s perception of these probabilities can be overweighted or underweighted. In certain situations, the agent could even be entirely insensitive to them. To model this, we define a function *w* that represents the “subjective probability weight” that assigns the agent to each outcome. When the agent is able to perceive probabilities without distortion, then *w*(*p_xi_*) = *p_xi_*, but, in general, it will not be the general case. Apart from it, the agent values the choices in terms of its utility function *U* assigning numerical values (“utilities”) to the outcomes {*U*(*a*_1_), *U*(*a*_2_), *U*(*b*_1_), *U*(*b*_2_)}, in such a way that outcomes with higher utilities are always preferred.

The agent will make a decision, *A* or *B*, computing an overall score associated to each decision and defined, in the case of decision *A*, as

$$\sigma \left(A\right)=w\left(p_{a_{1}}\right)\cdot U\left(a_{1}\right)+w\left(p_{a_{2}}\right)\cdot U\left(a_{2}\right)$$

and similarly for *B*,

$$\sigma \left(B\right)=w\left(p_{b_{1}}\right)\cdot U_{1}\left(b_{1}\right)+w\left(p_{b_{2}}\right)\cdot U_{2}\left(b_{2}\right)$$

σ(*A*) and σ(*B*) estimate the “expected value” of each of the option. The agent selects the option with the highest value. Only in rare situations will the utility function *U*(*x_i_*) for every outcome and the values of the probabilities {*p_a_*_1_, *p_a_*_2_, *p_b_*_1_, *p_b_*_2_} be fully known in advance. In general, there are several methods to estimate these probabilities and to model utility functions (e.g., Ravichandran and Baker, 1989).

For the case of dual systems, the expected value associated to each possible decision is a combination of the values assigned to it independently by the System 1 and System 2 (Mukherjee, 2010). In the previous example, where a decision has to be made between two choices *G* = {*A*, *B*}, the agent’s decision is the result of a rational σ*_R_*(*G*) and an emotional σ*_E_*(*G*) subsystem that are combined to produce a global measure σ(*G*).

Each subsystem assigns an independent value on the basis of its own method of evaluation (Hsee and Rottenstreich, 2004): each one will have a different probability weighting function whose value will depend on the affective nature of the outcomes and the sensibility to them.

One way of combining both subsystems is as a weighted sum:

$$\sigma (G)=\alpha \cdot \sigma_{R}(G)+(1-\alpha )\cdot \sigma_{E}(G),\text{ where G}=\text{\{}A,B\text{\} and }\alpha \leq \text{1}$$

In general, the weight α will be affected by different factors: history, dispositions, nature of the outcomes, nature of the task, temporal proximity between decisions, etc., and σ*_R_*(*G*) and σ*_E_*(*G*) will be calculated according to previous equations for the values *G* = {*A*, *B*}.

In these models, the final output is a combination of the evaluations of the each system. This formulation does not necessarily imply that the agent uses both systems in each given situation. It provides a statistical description over a large enough sample of decision events that is also valid if we assume that only one of the two systems will drive the decision-making at each moment.

In all these cases, the value of an option is based only on the local expected utility of the outcome. However, the existence of non-linearities and feedback loops can often undermine this assumption. For such situations, for instance, for the consideration of multiple attributes in the options that require different attention, we must consider non-additive interactions in decision processes and analyze the resulting models from a dynamical systems perspective. Such is the case of, for instance (Regenwetter et al., 1999) or models within the Dynamical Field Theory (DFT; Busemeyer and Townsend, 1993; Roe et al., 2001; Busemeyer and Johnson, 2004). Although the DFT was designed to account for findings from risky decision-making (Busemeyer and Townsend, 1993), a multi-attribute decision-making version (Diederich, 1997) and most recently, a multi-alternative choice behavior model (Roe et al., 2001) have also been developed. In DFT the utility of the options is not evaluated independently of each other but rather they are compared along their attributes. The probability of making a specific choice varies according to which attributes receive the agent’s attention during decision-making. Preferences over the options continue to evolve over time until the agent’s inclination for one of the options becomes strong enough to exceed a threshold. The corresponding alternative is then chosen.

DFT provides a formal description of the “dynamic evolution of preferences” during deliberation. Psychologically, the fluctuations in the decision-maker’s attention to attributes and states over time represent “doubts,” changes in the agent’s opinion before making a decision, etc. Formally, DFT is modeled by a Markovian process based on a quantitative preference state. Choice probabilities are calculated by means of the diagonalization of the system and this determines the stationary probability over the stochastic process (Busemeyer et al., 2006) and subsequently the states toward which the system evolves.

DFT accounts for the evolution of preferences during a single decision event. Once a decision is made, the agent encounters a similar situation to the previous one, i.e., another independent dynamical process starts for the next decision. DFT, therefore, also considers global decision chains as a sequence of separate events whose outcomes are obtained dynamically and in which the only dynamical interactions taken into account are internal to the agent, rather than being interactions between internal and ecologically embedded processes, as for example, the case of a medical treatment over an extended time-course.

The model presented in the next sections applies Markov chains to a dynamical dual system situation. It differs from how these are generally applied (Scheibehenne et al., 2009) in that it accounts for interactions between different decision events (going beyond DFT, but using some of its formal methods). We study the effects of such non-linear, time-extended interactions on the function of the dual system architecture. It is to be expected that in this case the diagonalization process will change their effective cognitive structure and relative weight between the emotional and rational components (the value of α). In fact, we find that the combination of the two systems cannot be expressed in linear terms any longer, thus leading to non-trivial effects that provide answers to the questions we have raised in the introduction.

## 3 Somatic Markers Hypothesis: A Mathematical Description

Before presenting our model we need to introduce a formalization of the SMH that uses minimal assumptions (see Cognitive Ability and Predictive Capability of an Agent in Appendix for technical details and definitions). The dual systems distinction between reasoning and emotions assumes a two-tiered model of decision-making processes (Sloman, 1996; Evans, 2003, 2008; Weber and Johnson, 2009). The SMH asserts that the function of the somatic markers (SMs) is to create domain-specific associations between a situation and primary emotional states, thus providing a link (secondary emotions) between past experiences and the current situation (which may be novel but still resemble previous experiences in some aspects).

The SMH divides decision-making processes into two groups: reasoning and emotions, primary and secondary (for details, see Table A1 of Cognitive Ability and Predictive Capability of an Agent in Appendix). We want to find parameters to characterize the ability of an agent engaged in decision-making to act correctly depending on whether it is guided by its own emotions or by a deliberative process or a combination of both.

To this aim, let us define first the notion of “predictive ability” of an agent. This can be used to characterize reasoning, primary, and secondary emotions.

- We define the *reasoning ability* *R* of an agent (and similarly the *primary emotional* and *secondary emotional* abilities) in a state *s* of the world Ω, as the capacity to correctly propose a particular action μ in a given situation s ∈ Ω using deliberative processes.
- We define the *“deliberative predictive ability*” of an agent as the probability of the action taken, μ, in a given situation s ∈ Ω being *correct*. By *correct* we mean that the action satisfies some viability constraint or maximizes some utility of interest to the agent. We denote this as *P_R_*(*s*, μ) and, likewise, we define $P_{E_{1}}$(*s*, μ), and $P_{E_{2}}$(*s*, μ) for the primary and secondary emotional abilities respectively.

**Figure 1:**
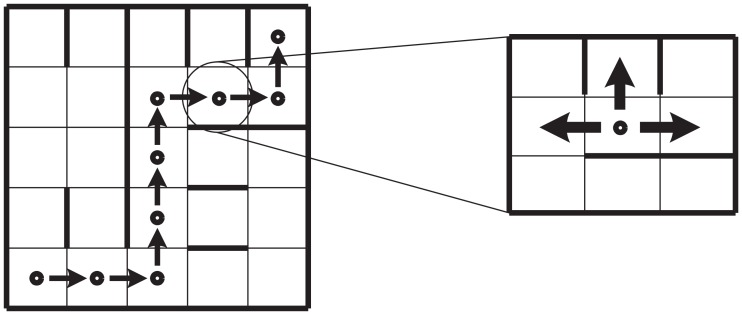
**Representation of an agent in a grid that must move from *s*_0_ to *s_f_***. The shortest path is shown (left). At every step, there exist one correct and one wrong choice and the option to move back to the previous state (right).

In general, the SMH is interpreted as a linear process. We discuss this in an example: an agent must move along the shortest path from initial position *s*_0_ to final position *s_f_* in a grid (Figure 1). At each step, the agent can: (i) follow the shortest path, (ii) make a wrong decision (not following the shortest path), or (iii) consider the current state as risky (potentially a mistake, whether it actually is one or not) and therefore go back to the previous position.

Let us suppose that along its way, the agent makes some mistakes (sometimes believing it is correct, other times stepping back after considering the new situation as dangerous). To quantify the optimality of the agent, we measure the adequacy of its behavior in terms of a confusion matrix (Kohavi and Provost, 1998).

This matrix consists of a two-class table that contains information about real situations (rows) and states predicted by the system (columns). Thus, the diagonal elements in a confusion matrix represent the correctly classified predictions according to the actual outcome while the cross-diagonal elements represent misclassified ones.

Our aim is to express, in terms of the confusion matrix, the predictive abilities introduced above. The matrix provides information about the performance, giving the number of situations from one class (positive/negative) classified into another (or same)class and represented by four coefficients with the following meaning: *a* is the number of correct predictions of a situation being positive, *b* is the number of incorrect predictions of a situation being negative, *c* is the number of incorrect predictions of a situation being positive, and finally, *d* is the number of correct predictions of a situation being negative.

**Figure 2:**
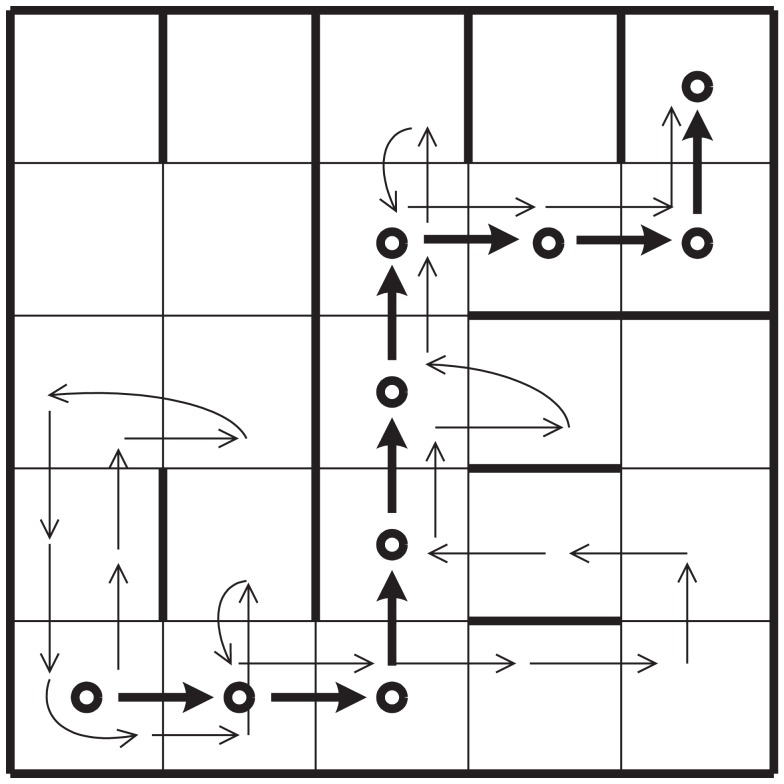
**Representation of a decision-making agent with reasoning and primary emotional abilities on a grid that must move from *s*_0_ to *s_f_***.

Let us consider an agent that goes from *s*_0_ to *s_f_*, in *n* steps. Let us suppose that the agent uses reasoning abilities when it is possible to act with absolute certainty but becomes completely cautious in the presence of uncertainty. In other words, the performance in this case is a mixture of the capabilities *R* and *E*_1_. Let us suppose that the path of the agent on its way to *s_f_* is the one shown in Figure 2. We can then derive from this which decisions have been positive and which ones have been negative. We can express this result in terms of the agent’s predictive abilities. In general, the probability of an event happening is identified with its frequency given a large enough sample.

We denote by (a*, b*, c*, d*) the ratio between absolute coefficients (*a*, *b*, *c*, *d*) divided by the total number of cases *n* = (*a* + *b* + *c* + *d*), then:

- The *accuracy* of the agent can be expressed as the proportion of the total number of predictions (positive, a*, and negative, d*) that are correctly classified.
- Similarly, the *inaccuracy* of the agent can be expressed as the proportion of the total number of predictions that are incorrectly identified (b*, c*) and it includes two type of errors: b* identifies the error of avoiding situations when they are suitable, while c* indicates a situation deemed adequate when in fact it is not.

In this way, we can identify in a confusion matrix (Table 1) the ability of the agent *X* to infer right decisions considered for all, *s* ∈ Ω, that it is denoted by *P_X_*, as the average of the right choices (a* + d*).

$$P_{X}=\left(a^{*}+d^{*}\right)$$

**Table 1:** **Two-class confusion matrix**.

		Prediction	
		Positive	Negative
Actual	Positive	a	b
	Negative	c	d

*P_X_* is a measure of the predictive capability of agent *X* (and similarly, 1 − *P_X_* = (c* + b*) is a measure of the incorrectness of the agent).

Although the SMH does not specify the interactions between the deliberative and emotional mechanisms at the moment of taking a concrete decision, what is often suggested is that depending on the situation, one system is dominant at a given time. This permits the identification of the frequency with which each mechanism is used (according to the definitions provided in Cognitive Ability and Predictive Capability of an Agent in Appendix) and in this way the quantification of the predictive abilities of each of the mechanisms on its own (*P_R_*, $P_{E_{1}},$ $P_{E_{2}}$). This means that when a system uses more than one mechanism we can model this by considering *P_X_* to be the weighted sum of the predictive abilities of each mechanism involved (reasoning, primary, and secondary emotions). In the case of an agent without secondary emotions, the predictive ability (measured over many trials) will be,

$$P_{X}=\alpha P_{R}+\left(1-\alpha \right)P_{E_{1}},\;\;\;\left(\alpha \leq \;1\right)\;$$

This can be interpreted as meaning that in an average “single decision-making” situation the agent applies its reasoning abilities or is guided by primary emotions according to the weight factor α. This factor is not a property solely of the agent, but of the agent in a given environment, since it depends on the information that the environment provides.

What is the effect of adding somatic markers to this picture? A “somatic marker” agent would make deliberative considerations (*R*) and would make use of primary emotions (*E*_1_) to respond to certain situations. In addition, it would also incorporate secondary emotions (*E*_2_) linking specific aspects of the situation and somatic states that provide a relevant response. As before, we maintain the assumption that one given mechanism is dominant at a specific situation. Therefore, averaging over many independent decision-making events, we obtain:

$$P_{X}=\beta P_{E_{2}}+\left(1-\beta \right)\left[\alpha P_{R}+\left(1-\alpha \right)P_{E_{1}}\right],\;\;\;\;\left(\beta \leq \;1\right)$$

It is easy to, see that, if we treat decision events as independent, the effect of $P_{E_{2}}$ on the overall system is positive only if it is greater than the predictive ability of the other two mechanisms combined. In other words, there is a positive overall effect of incorporating somatic markers only if

$$P_{E_{2}}>\alpha P_{R}+\left(1-\alpha \right)P_{E_{1}}$$

The condition on $P_{E_{2}}$ seems intuitively correct and is compatible with the interpretation that somatic markers serve to complement reasoning and primary emotions in decision-making. It would appear that the only way in which somatic markers make sense is if their predictive ability surpasses the combined predictive ability of reasoning and primary emotions. Although this seems a logical consequence of the SMH, we will show it not to be correct in general: it is based on the linearity of the framework in which the assumptions have been stated, in particular the assumed independence of decision events used in the averaging process.

In the SMH formulation, Damasio assumes an oversimplified framework in which the effects of incorrect predictions based on wrong emotional markers are evaluated without taking into account the complex world of opportunities and the unexpected situations that typically characterize our daily life. As a contrast to this typical interpretation of the SMH we will show that a dynamical model is able to better capture the non-linear aspects of real life decision-making leading to non-intuitive results.

## 4 A Non-Linear Model of the SMH

The above interpretation of the SMH assumes that, when making a choice, the value of an option is based only on the locally expected utility of its outcome. In this section, we maintain the assumption of local mechanism dominance (one subsystem dominates a local decision event), and model the predictive abilities for each mechanism exactly as before. However, we apply these definitions to a non-linear framework where decisions may interact non-additively.

**Figure 3:**
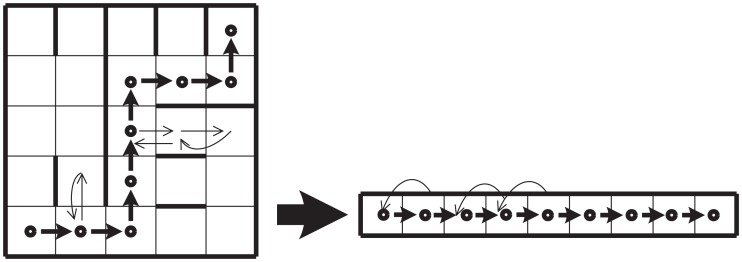
**Discrete-time Markov chain representing the decision process of an agent**.

**Figure 4:**
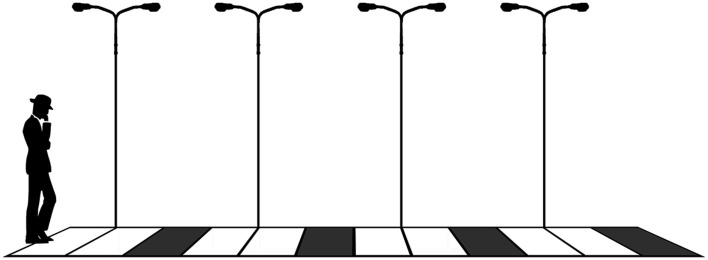
**Illustration of the example of the man going on the road to his home**.

We examine the effects of modeling decision-making as a Markov chain using our grid example. We first study a one-dimensional situation (Figure 3). To help interpret this scenario, consider a man who is returning home at night walking along a country road. The road leads directly to his house and there are no bifurcations (Figure 4). The goal is to reach the house. However, the road has not been well maintained and there is the danger of stumbling on debris or stepping into a pothole on the ground. There are lampposts on the road, but not many, so that there are segments where there is no light to help the man see where the next step should fall. Every now and then a car passes by and with its headlights illuminates the spot of the road where the man is walking.

We imagine that the man proceeds by a series of local decisions on whether to move ahead or step back to make sure that the next step will not result in a fall. If there is enough light, the man makes a rational decision to move forward (avoiding any possible danger that the can see). If it is dark, the man will most likely take a step to a spot with more light and wait for a car to pass by and shine a light on the area ahead. We consider that being able to see the ground where the next step should fall triggers a decision that is mostly dominated by the deliberative capacity, while the cautious attitude taken in dark conditions is mostly the result of primary emotions.

These decisions are probabilistic. For instance, in the dark, primary emotions normally recommend caution and stepping back into the light, but occasionally they result in taking a step forward anyway. Conversely, when the road is illuminated, the deliberative capacity sometimes does not reach the rational conclusion to move ahead, in spite of the information available (i.e., deliberative capacities are not perfect). This signifies that *P_R_* will be a number close to 1 but not 1 (a high probability of making a choice that advances toward the goal) and $P_{E_{1}}$ will be a number closer to 0 but not 0 (a low probably of advancing when primary emotions dominate).

We consider first a case without secondary emotions. In a one-dimensional setting, mistakes due to fear or caution either caused by primary emotions, or by a faulty deliberative mechanism are both represented as a step backward in the chain (see Figure 3). What is important about this example is that we are not dealing with a single instance of decision-making but with a chain of many decisions extended over time. The resulting formal structure will be applicable to more general situations. In order to explore the example quantitatively we analyze a particular case that clearly illustrates what we mean by non-linear, non-additive interactions between decision events.

Let us suppose in the first instance that the road leading the man to his home is made up by a series of discrete cells (as shown in Figure 4) and that whether the man will make a decision dominated by his rationality or by his primary emotions depends solely on whether the cell is illuminated or dark. When there is light the man is likely to evaluate the situation clearly and decide to advance (avoiding dangers if there were any). This is equivalent to deciding to advance with probability equal to his rational predictive capacity *P_R_*. The situation is different when the cell is dark. The man relies on primary emotions and exercises caution. In this case, the probability of advancing is $P_{E_{1}}$ (which is much lower).

These assumptions mean that the effective distribution of exercises of rational or emotional decision-making depends solely on the distribution of light and dark cells. Calculating, as before the combined probability of advancing as

$$P_{X}=\alpha P_{R}+\left(1-\alpha \right)P_{E_{1}}$$

as if decisions were independent of each other would be equivalent to saying that α simply represents the proportion of cells with light. For reasons of simplicity, let us assume a regular distribution of two cells with light followed by one dark cell (Figure 4 the non-additive interactions that are central for our results remain present for any distribution of dark cells).

**Figure 5:**
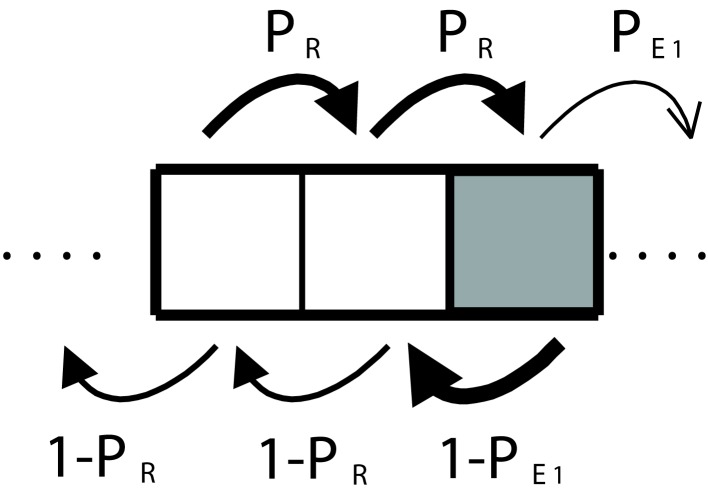
**Discrete-time Markov chain corresponding to a three-step sequence**. In the white cells, the agent uses the deliberative subsystem and primary emotions in the dark gray ones.

Let us also assume that the road is sufficiently long, so that as we describe the transitions between states using a discrete Markov chain, the distribution of probabilities will settle into a stationary state (see A Formal Framework to Model Dynamical Dual Systems in Appendix). Assuming that, on average 2/3 of the time, the agent will make a decision using subsystem *R*, and 1/3 of time subsystem *E*_1_, we can reduce the analysis of the dynamics of a three-step transitions process that repeats itself (Figure 5), We now ask: what is the “effective” decision-making architecture in the stationary condition? What are the relative weights of the two systems? Are they still given by the proportion of cells α? While the environment remains a strong factor, the answer cannot simply be that the relative weight between the subsystems is given by the distribution of dark and light cells (α) since we must also take into account the *frequency* with which the man visits each kind of cell and this frequency in turn depends on the effective probabilities of transitions between cells, making this a recursive problem. Dark cells are harder to go through and tend to produce a “trapping effect” until eventually, the man risks moving forward with a probability equal to $P_{E_{1}}.$ The light cells just before a dark one will then tend to be visited more often than one third of the times and so will the dark cells.

To put a numerical example (see A Formal Framework to Model Dynamical Dual Systems in Appendix for details) if $P_{E_{1}}=0.1$ and *P_R_* = 0.8, instead of a flat distribution of 1/3 of visits each, dark cells are visited 40% of the time, the light cell just before a dark one receives 47% of the visits and the light cell just after a dark one receives 13% of the visits. Notice the trapping effect that makes the man spend more time crossing the boundary between the light cell and the adjacent dark one (see arrows in Figure 5). This means that the man uses rational decision-making 60% of the time and not 66.66% as would be expected from the normal assumption of linear interactions between decisions. Similarly, primary emotions are used 40% and not 33.33% of the times.

While the numerical values may differ with non-regular cell distributions, we suggest that the effects are likely to be even more pronounced in such cases since a regular distribution puts as much space as possible between the dark cells – the ones that produce the trapping effect. Having regions of dark cells with less or no separation is likely to make the trapping effect even stronger. Similarly, we suggest that the non-linear effects would also be present if the factors determining whether the agent exercises rational or emotional decision-making are not fully given externally, but depend on internal conditions as well.

Our first result indicates that *in non-linear decision chains primary emotions weigh more than expected*. This result directly affects the system’s predictive capability *P_X_*. Expressed in terms of the initial architecture of the agent, the intuitive linear interpretation can lead us to wrongly consider the summation performed should be arithmetic,

$$P_{X}=\;\overset{2}{\underset{i=1}{\sum }}\frac{1}{3}\cdot P_{R}+\frac{1}{3}\cdot P_{E_{1}}$$

however, we must calculate *P_X_* in terms of the probability in the stationary state, once the visiting frequencies to each situation have settled. Substituting the values *P_R_* = 0.8 and $P_{E_{1}}=0.1,$ and using the stationary probability distribution, the predictive ability of the agent will be *P_X_* = 0.52 and not $P_{X}=\left(\frac{2}{3}\cdot P_{R}+\frac{1}{3}\cdot P_{E_{1}}\right)=0.56.$

In order to examine the effect of secondary emotions in a similar vein, we consider that SMs work by sometimes overriding deliberative capabilities and primary emotions. In the example, when the man is in a dark spot, for instance, the action of SMs could make him advance toward his goal even when primary emotions recommend staying put. This may be due to previous experience with the overall situation (which does not necessarily relate with relevant information for the task, e.g., a gust of fresh air may encourage the man behave more bravely).

Similarly, even under the light of a lamppost when all the information available should trigger a rational decision to step forward, an aspect of the situation (again not necessarily connected to the task) may trigger secondary emotions that recommend caution (e.g., the play of shadows ahead evokes an unpleasant, but irrelevant, memory). In the first case the effect of secondary emotions would be positive (the man advances toward the goal) and in the second case negative. This is equivalent to saying that SMs can make the man advance toward his goal with probability $P_{E_{2}},$ which will be a number between $P_{E_{1}}$ and *P_R_*.

**Figure 6:**
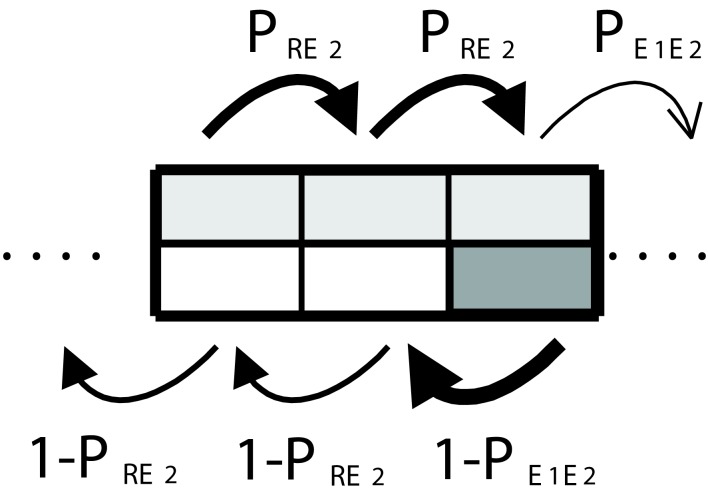
**Somatic marker system**. The use of somatic markers (50% of the time in average) is represented by light gray areas that take up the half of the cells.

As before, we assume that the illumination in a cell determines whether the man will advance with probability *P_R_* or with probability $P_{E_{1}}.$ In addition, we assume as a particular case that in both kinds of cells SMs will override the local decision-making mechanism half of the times. Graphically this is depicted in Figure 6. The light gray color in each cell indicates that the man will use secondary emotions 50% of the time (this is equivalent to choosing parameter β = 1/2). As before, the stationary distribution of transition probabilities between cells depends on the frequency with which these are visited. This involves a recursive problem and the solution is guaranteed to exist if the system fulfills the Markov property (see A Formal Framework to Model Dynamical Dual Systems in Appendix).

Damasio takes it as obvious that “defective” SMs (inaccurate linkages between emotional experiences and situations) are unlikely to be adaptive (Damasio, 1996). As far as we know, this statement has never been subsequently put into question, although no attempt has been made at producing a quantifiable operational version that could be put to the test. Using the formalism introduced in the previous section, locally “defective” SMs are those that result in a predictive capacity that is lower than the combined predictive capacity of reasoning and primary emotions. The effect of these secondary emotions in an isolated decision event will tend to be negative on average. The question is whether the global effect remains also negative. To calculate this we need to consider the possible interactions between decision events.

The general view is that uncertainties and mistakes always play a negative role. However, when decision events are not independent, making a mistake locally could remedy a previous mistake. Should we in this case compute two mistakes or one right decision instead?

We can now ask at what point secondary emotions start to have a positive overall effect. As we mentioned, we have established in the previous section that in the case of independent decision events, secondary emotions only have a positive effect if

$$P_{E_{2}}>\alpha P_{R}+\left(1-\alpha \right)P_{E_{1}}$$

Does the same condition apply in situation of non-additive interactions? In our example, it seems clear that any decision system acting in isolation will have a positive effect if it gives the agent a probability larger than 1/2 of advancing toward the goal. This is valid in the general case. A baseline decision-making system is one where the number of correct and incorrect predictions is the same: a* + d* = c* + b* (see Table 1).

The predictive ability of such a system is therefore 1/2. We are interested in the case in which the agent’s predictive abilities are defective, i.e., *P_X_* < 1/2 (in terms of a confusion matrix, we would have c* + b* > a* + d*, that is, agents that are wrong most of the time).

Let us consider the SM agent (see Figure 6) using in equal proportion (50%) predictive abilities $P_{RE_{1}}$ and $P_{E_{2}}.$ The benefits of including correct SMs can be also shown in relation to the baseline but they strongly depend on whether we adopt the non-linear analysis or not. For example, if $P_{RE_{1}}<1∕2$ (for instance, with *P_R_* = 0.55 and $P_{E_{1}}=0.05,$ so $P_{RE_{1}}=0.34$), and somatic markers resulting in $P_{E_{2}}=0.6$ are added, we obtain following a linear approach that the total combined predictability is $P_{RE_{1}E_{2}}=0.48,$ while if we take a non-linear approach $P_{RE_{1}E_{2}}=0.51$; in other words, going from below to above the baseline (see A Formal Framework to Model Dynamical Dual Systems in Appendix).

We are interested in exploring whether assembling defective components $\left(P_{E_{2}}<1∕2\right)$ to build the emotional-cognitive architecture of an agent that already performs below the baseline $\left(P_{RE_{1}}<1∕2\right)$ can produce a reliably favorable effect ($P_{RE_{1}E_{2}}\;>$ 1/2). Moreover, we will also ask $\left(P_{E_{2}}<P_{RE_{1}}\right).$ Under such circumstances, as we have seen based on the linear analysis, it should not be possible for SMs to have any overall positive effect. Decisions made by SMs should at the local level lead to even more mistakes than decisions made with the combined rational/primary emotional system. However, we find in the non-linear analysis (see A Formal Framework to Model Dynamical Dual Systems in Appendix) that in spite of the negative conditions on $P_{E_{2}}$ and $P_{RE_{1}},$ the combined system can result in *P_X_* > 1/2.

**Figure 7:**
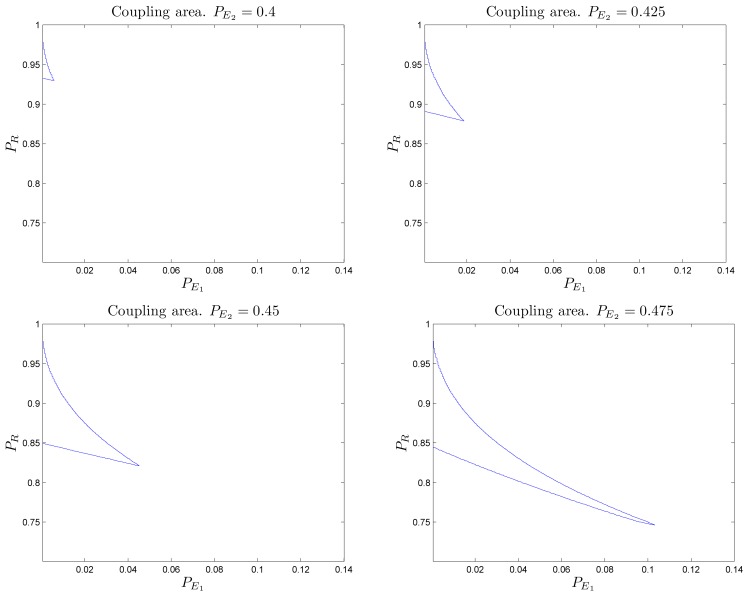
**Different regions in the parameter space $\left(P_{R},P_{E_{1}}\right)$for which wrong somatic markers cooperate with a “bad” reasoning/primary emotional system to form a decision-making system with better than baseline behavior**. Values for the regions illustrated are: (upper left) $P_{E_{2}}=0.4;$ (upper right) $P_{E_{2}}=0.425;$ (bottom left) $P_{E_{2}}=0.45;$ (bottom right) $P_{E_{2}}=0.475.$ The size of the regions increases as $P_{E_{2}}\to 1∕2.$

Figure 7 shows the regions of parameter space within which the resulting combined system behaves above the baseline for different values of “defective” SMs. We present analytical results and numerical simulations of the conditions under which this counterintuitive effect occurs for the particular set of parameters used in our example (α = 2/3 and β = 1/2 selected for analytic convenience).

**Figure 8:**
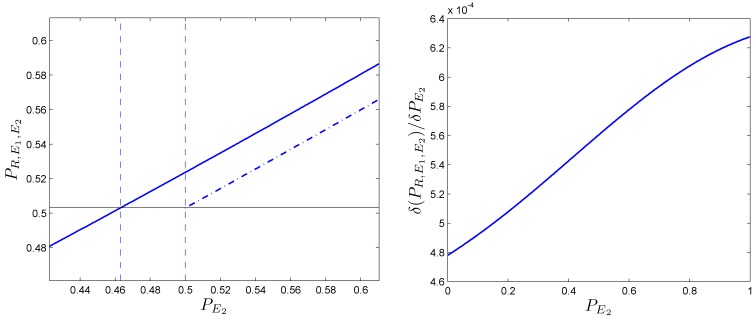
**Robustness of the phenomenon in which coupling is positive**. (Left side): evolution of the predictive ability *P_X_* of the somatic marker agent when $P_{E_{2}}$ is increased. It is compared the non-linear case (solid line) vs. the linear case (dotted line). Besides the trivial increasing line, the function is slightly curved. (Right side): its derivative function with respect to $P_{E_{2}},$ denoted by $\frac{\delta \left(P_{R}E_{1}E_{2}\right)}{\delta \left(P_{E2}\right)},$ allows us to observe the effect enlarged.

We now study the robustness of this phenomenon. If we plot the dependency of $P_{RE_{1}E_{2}}$ with respect to $P_{E_{2}}$ (Figure 8-left), it can be shown that the function is not a straight line but is slightly curved. This effect can be analyzed if we calculate the derivative function of $P_{RE_{1}E_{2}}$ with respect to $P_{E_{2}}.$ This is also represented (see Figure 8-right); three observations can be made:

• In the first stage, for values of $P_{E_{2}}\in \left(0,\;0.3\right),$ $P_{RE_{1}E_{2}}$ is less than 0.5.
• In the second stage, for increasing $P_{E_{2}}$ values, $P_{RE_{1}E_{2}}$ also increases.
• In the third stage, for values of $P_{E2}≳0.8,$ the effect on $P_{RE_{1}E_{2}}$ starts to plateau.

If we focus on the second stage, we can conclude that the improvement for the whole system is not a linear function of secondary emotions. From this we could infer that an improvement in $P_{E_{2}}$ would be better for the global system’s behavior than an improvement in the deliberative ability of the system, *P_R_*. We can deduce that when the deliberative capacity is sufficiently high $\left(P_{R}≳0.8\right),$ incrementing the value of *P_R_* generates only a small increase in the predictive ability of the somatic marker agent. In other words, for similar conditions, increasing the deliberative capacity of an agent *RE*_1_ is less efficient than increasing $P_{E_{2}}$ in a somatic marker agent (*RE*_1_*E*_2_).

### 4.1 Extending the result to two dimensions

**Figure 9:**
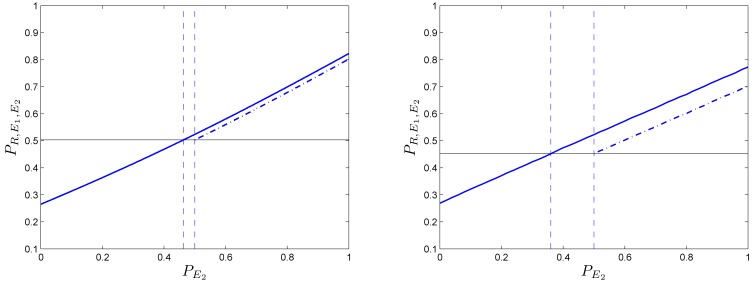
**One-dimensional (left column) and two-dimensional (right column) phenomena of positive coupling**. (Left side): in the first plot, the predictive ability $P_{RE_{1}E_{2}}$ is represented ($P_{E_{1}}=0.1,$ *P_R_* = 0.8). The linear case in dotted line, the non-linear case in solid line. (Right side): it can be noticed that the two-dimensional example requires lower values of $P_{E_{2}}$ to obtain similar outcomes than in the one-dimensional one. All values have been obtained after 50,000 simulation runs.

The results obtained for one-dimensional Markov chains can be generalized for grids of higher dimensions. We examine the two-dimensional case using numerical methods. Figure 9-left shows the probability $P_{RE_{1}E_{2}}$ of an agent in a two-dimensional grid (assuming $P_{E_{1}}=0.1$ and *P_R_* = 0.8).

The effect is slightly more marked than the one for the one-dimensional problem (Figure 9-right) because positive couplings emerges at lower values in the process. In order to further study the relation between the deliberative system and the secondary emotional system, we fix $P_{E_{1}}=0.1,$ and find the pairs of values (*P_R_*, $P_{E_{2}}$) for which and agent with secondary emotions starts to do better than without them.

**Figure 10:**
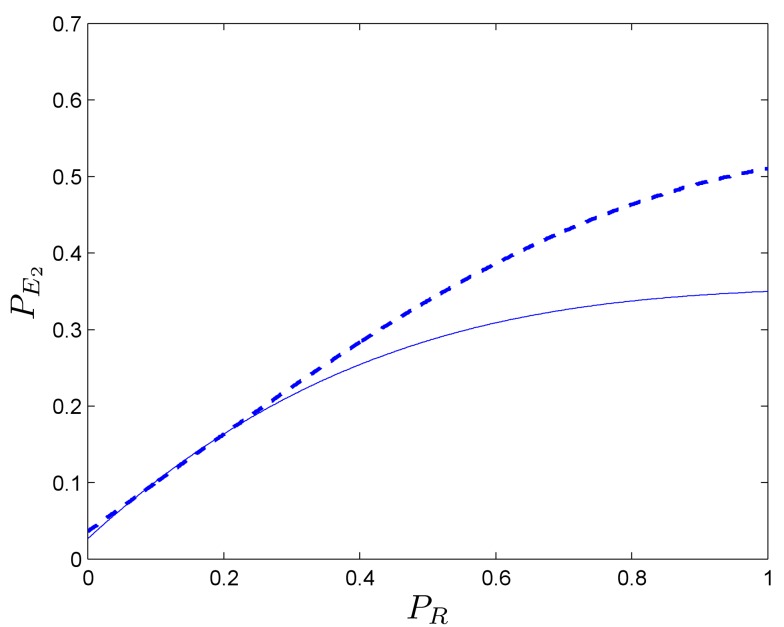
**In a one-dimensional case, the evolution of the bordering value (solid line) of those regions where the coupling phenomenon appears is presented (with $P_{E_{1}}=0.1$)**. It can be seen that, in the two-dimensional case (dashed line), the positive coupling emerge with values of $P_{E_{2}}$ lower than the ones in the one-dimensional case. All values have been obtained after 10,000 simulation runs.

Figure 10 shows pairs of values (*P_R_*, $P_{E_{2}}$) at which a positive coupling occurs in one (solid line) and two dimensions (dashed line). It can be seen that for high values of *P_R_* (i.e., $P_{R}≳0.6$):

• The size of positive coupling regions with $P_{E_{2}}<1∕2$ is larger than in the one-dimensional case.
• The effect of the positive coupling arises at lower values than in the case of $P_{E_{2}}.$

Again we conclude that for high *P_R_* values, no considerable improvements for the somatic marker agent are derived from the enhancement of its deliberative capacity. Rather, its performance depends more strongly on $P_{E_{2}}.$ According to these results, increasing the dimensions of the problem from one to two, favors the positive coupling effect of “wrong” secondary emotions, allowing the agent a larger margin for inaccurate somatic markers that lead to an overall positive effect.

## 5 Discussion

Our non-linear model has uncovered two empirically relevant implications of the SMH when applied to decision chains. These implications are unintuitive within the picture of decision-making as an isolated event. Without making any strong assumptions about the corresponding mechanisms other than their predictive ability, we first notice that the *local* frequency with which each of the three mechanisms takes the lead role (parameters α and β in our model) does not correspond to the *effective* weight of each mechanism on the overall decision chain. This is clear evidence of non-additive interactions between decision events.

The analysis has been performed for decision chains in the absence of very costly (e.g., potentially lethal) outcomes. In these cases, the cautionary effect of primary emotions tends to be over-represented and reduces the effectiveness of the deliberative predictive ability. This result in agents that can be overcautious along a decision chain, over and above the degree prescribed by their primary emotions in an isolated decision event.

The second result shows the apparent paradox that the combination of “bad” mechanisms can lead to good (better than baseline) decision-making. How is this possible? It is easy to notice that although $P_{E_{2}}$ is worse than $P_{RE_{1}},$ when mixed, subsystem *E*_2_ can break up the emotional blocking effect generated by system *RE*_1_. In other words, the combination breaks the over-cautionary effect of primary emotions. In short, somatic markers act as unblocking mechanisms that force agents to leave those states in which they are stuck by the over-representation of primary emotions.

The phenomenon is only apparently paradoxical. Non-linear stochastic systems are known to behave in unexpected and counterintuitive ways (as, for instance, in discrete-time Brownian rachets or Parrondo's games, Parrondo et al., 2000). In many cases, stochasticity can play a role in stabilizing the combination of unstable systems (Reimann, 2002), resulting in effects documented in biochemical enzyme transport (Westerhoff et al., 1986), financial processes (Maslov and Zhang, 1988), and population genetics (McClintock, 1999). More directly relevant to our results, similar effects have been found in gambling games where the right combination of losing strategies results in a positive expectation of winning (Parrondo and Dinis, 2004).

Explanations of this phenomenon in information-theoretic terms (Harmer et al., 2000) or in terms of signal-to-noise ratio (Fuh and Yeh, 2001), indicate that the paradox “*losing* + *losing* = *winning*” is simply a version of the well-known effect *“chaos + chaos = order”* in non-linear dynamical systems (Harmer et al., 2002; Parrondo and Dinis, 2004).

The results indicate the presence of a similar phenomenon in the interaction between a combined reason/primary emotion system with a tendency to get stuck in the advance toward the goal and a somatic marker system with lower than baseline predictive ability. The synergistic overall effect is positive and above baseline, indicating that mutual (partial) cancelation of the two negative subsystems. In contrast to Damasio’s interpretation of SMs, the factor determining the overall advantage of secondary emotions in decision chains is not their predictive ability, but the structure of non-linear relations between the three mechanisms: reasoning, primary, and secondary emotions, which of course, are all environment-dependent.

## 6 Conclusion

We have questioned some of the basic assumptions that underlie conceptual and empirical work on dual system approaches to decision-making and we have expressed an alternative in a formalization and model of the SMH. It is clear that common sense intuitions such as assuming that SMs must have a positive predictive ability to make sense are found wanting when applied to scenarios involving stochastic, non-independent decision chains. This is even the case if we keep the Markovian assumption and consider decision-making and actions as discrete events. We can expect the effects to be possibly more marked if there is a deeper dependence on history or if decisions and actions combine in more complex ways across continuous timescales since the possibilities for synergistic couplings would be enlarged.

Our result is restricted to the analysis of the model for one set of parameters (α and β chosen to facilitate the analysis) in one dimension and the numerical confirmation of a larger effect in two dimensions. As we are interested in providing an existence proof showing that SMs need not have a local positive effect to be useful, it is not central to the aims of this paper to explore the behavior of our model for the full range of α and β, although it would be straightforward to examine this numerically. Similarly, it is possible that the effect is even more pronounced in higher dimensions corresponding to situations with more local options on average. While these extensions would complete the picture, the very existence of cases that contradict the assumptions about the benefits of SMs is the central message of this study.

We have tested the role of SMs in decision chains for situations that do not involve very costly negative outcomes. It is to be expected that primary emotions will nevertheless tend to recommend caution if environmental circumstances resemble risky situations. This is contemplated in our model by assuming low values for $P_{E_{1}}.$ What is unexpected is that this cautionary effect can be amplified by the interactions with other decision events, resulting in decision-makers “getting stuck” along their path and decreasing the impact of their deliberative predictive ability.

The fact that an experience-dependent affective subsystem may have evolved to enhance decision-making is given a new meaning in the light of our results. It is not necessary to put too strict a condition on the predictability of secondary emotions for their presence to start benefiting the agent. At any given point, decisions taken based on secondary emotions may lead to bad outcomes (less than baseline predictability) and yet the overall effect on the chain still remain positive. This gives the system a chance to adjust and improve with experience, resolving the problem of how SMs get their local positive functionality $\left(P_{E_{2}}>1∕2\right)$ during development in the first place. The coupled system that includes even defective or not properly adjusted SMs can still make the agent reap the benefits of using secondary emotions. In turn, it allows the agent to keep accumulating novel experiences necessary to refine its SMs. The initial functionality of secondary emotions would seem to be the encouragement of exploratory behavior. Moreover, we also have found that in agents with high predictive capability, increasing the efficacy of the deliberative capacity produces a decreasing marginal gain in comparison with the effect of better attuned SMs. This result suggests that for many environments, as an evolutionary strategy involving developmental plasticity, SMs may out-compete the evolution of sophisticated deliberative capabilities. This is due, on the one hand, to their weak dependence on their initial adjustment (they accrue positive effects even for local predictability below the baseline) and, on the other, to the fact that the benefits of increasing their adjustment during lifetime can be higher than developing more reasoning power. In view of these implications, the evolutionary plausibility of the dual-process accounts is strengthened by considering decision chains as a non-linear process.

## Conflict of Interest Statement

The authors declare that the research was conducted in the absence of any commercial or financial relationships that could be construed as a potential conflict of interest.

## Figures and Tables

**Figure A1:**
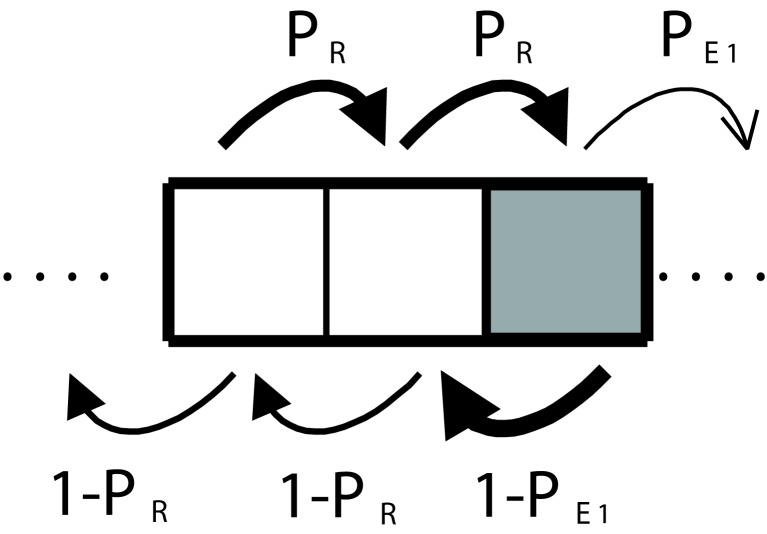
**A coupled (dual) agent-environment modeled through a Markov chain**.

## Appendix

### Cognitive ability and predictive capability of an agent

Here we express in formal terms some of the notions used to formalize dual system decision-making.

#### Cognitive ability

Let *X* be a decision-making agent, let *A* = {μ_1_, μ_2_, …, μ*_n_*} a set of possible actions, and Ω = {*s*_1_, *s*_2_, …, *s_m_*} a set of states of the world. We define the cognitive ability *C* of the agent, in a state *s* of the world Ω, as a mapping that drives the agent to propose an action μ in the given situation s ∈ Ω.

$$\begin{array}{cccc} C: & \Omega & \to & A\\ & s & \to & C\left(s\right)=\mu \end{array}$$

For more details (reasoning and emotional abilities), see Table A1.

**Table A1:** **Mathematical definitions for the main elements in the somatic marker theory**.

**REASONING ABILITIES**	
In decision-making, reasoning abilities *“imply that the decider has knowledge about the situation which calls for a decision, about different options of action (responses), and about consequences of each of those options (outcomes); [they] also usually imply that the decider possesses some logical strategy for producing valid inferences”* (p. 166). This conception of rationality relies on hypothetical optimal conditions of time availability, i.e., deciding well also means *“deciding in a time frame deemed appropriate for the problem at hand”* (p. 169), and counting on enough working memory	Let *X* be a decision-making agent, let *A* = {μ_1_, μ_2_, …, μ*_n_*} a set of possible actions, and Ω = {*s*_1_, *s*_2_, …, *s_m_*} a set of states of the world. **Definition:** The reasoning ability is a mapping R, $\begin{array}{l} R:\Omega \to A\\ \;s\to R(s)=\mu \end{array}$ only for a certain set of states, *s* ∈ *S_R_* ⊂ Ω. *S_R_* represents cases which neither involve an immediate action nor a survival response. We consider situations where the agent has enough time to deliberate and assign subjective values to his preferences
**PRIMARY EMOTIONS**	
Primary emotions are preorganized mechanisms that activate links between stimulus and responses in a fast automatic way, without explicit knowledge or a reasoning strategy. In Damasio’s own words: *“Not all biological processes which culminate in a response selection belong in the scope of reasoning and deciding”* (p.166); *“[…]we are wired to respond with an emotion, in preorganized fashion, when certain features of the world in our bodies are perceived, alone*, *or in combination”* (p. 131)	**Definition:** Let us call the primary emotions ability, denoted by *E*_1_: $\begin{array}{l} E_{1}:\Omega \to A\\ \;s\to E_{1}(s)=\mu \end{array}$ defined only for a set of states of the world $s\in S_{E_{1}}=\left\{s_{1},\;\ldots ,s_{m}\right\}\subset \Omega .$ $S_{E_{1}}$ refers to states that demand immediate actions for survival. The aim of *E*_1_ is to map each element with to a protective response. This will act as an alarm bell mechanism
**SECONDARY EMOTIONS**	
Secondary emotions are built gradually on the foundations of the feeling of primary emotions in connection to the object that excited it. They somehow link object and emotional body state. Damasio explains the meaning of the secondary emotions’ mechanisms as follows: *“[They] occur once we begin experiencing feelings and forming systematic connections between categories of objects and situations, on the one hand, and primary emotions, on the other”* (p. 134). He later speculates: *“[…]One of the advantages of “feeling” your emotional reactions is that you can generalize your knowledge, and decide, for example, to be cautious with anything that looks like [an object or situation that demands caution]”*(p. 133)	**Definition:** We define “emotional memory *M*” as a link between a couple state-action (*s*, μ) and *v* ∈ [0, 1], i.e., the *value* of that action in that state. $\begin{array}{l} M:\Omega \times A\to [0,1]\\ \;(s,\mu )\to M(s,\mu )=v \end{array}$ Let us call ability of secondary emotions *E*_2_: $\begin{array}{l} \;E_{2}:\Omega \times M\to A\\ (s,(s^{\prime },\mu^{\prime },v^{\prime }))\to E_{2}(s,s^{\prime },\mu^{\prime },v^{\prime })=\mu \end{array}$ which establishes an equivalence between an state *s* ∈ Ω, and a list of parameters (*s*′, μ′, *v*′) according to previous emotional experiences of the agent. This allows to choose an action μ in the state *s* ∈ Ω based on the relationship between (*s*′, μ′, *v*′)

*Verbal definitions on the left column, quotes from Damasio (1994) and mathematical formalization of the associated cognitive abilities on the right one*.

#### Predictive capability

Let *X* be a decision-making agent with cognitive ability denoted by *C*, let *A* = {μ_1_, μ_2_, …, μ*_n_*} a set of possible actions, and Ω = {*s*_1_, *s*_2_, …, *s_m_*} a set of states of the world. We define the predictive capability of a cognitive agent as the probability of the action taken, μ, in a given situation *s* ∈ Ω being *correct* (denoted by μ*). By *correct* we mean that the action satisfies some viability constraint or maximizes some utility of interest to the agent. We denote this as *P_X_*(*s*, μ) or, in general, *P_X_*.

In mathematical terms, it can be expressed as (see Table A2 for more details),

$$\begin{array}{cccc} P_{X}: & \Omega \times C & \to & \left[0,1\right]\\ & \left(s,C\left(s\right)=\mu \right) & \to & P_{X}\left(s,\mu \right) \end{array}$$

where *P_X_*(*s*, μ) represents the agent’s estimation of having a correct outcome from performing μ on *s* (i.e., it is the probability of the action taken, *C*(*s*) = μ being the correct one, μ = μ*).

**Table A2:** **Mathematical definitions of predictive (deliberative, primary, and secondary) capabilities measuring the probability of the action taken being correct**.

By using the mappings *R*, *E*_1_, and *E*_2_, as have been defined in Table 1, we introduce the following definitions. **Definition:** In mathematical terms, the deliberative predictive ability can be defined as,
$\begin{array}{l} \;P_{R}:\Omega \times R\to [0,1]\\ \left(s,R(s)=\mu )\to P_{R}(s,\mu \right) \end{array}$
*P_R_*(*s*, μ) represents the agent’s estimation of having a correct outcome from performing μ on *s* by using its reasoning abilities (i.e., it is the probability of the action taken, *R*(*s*) = μ, being correct μ = μ*). **Definition:** The primary emotional predictive ability, denoted by $P_{E_{1}},$ is defined as:
$\begin{array}{l} \;P_{E1}:\Omega \times E_{1}\to [0,1]\\ \left(s,E_{1}(s)=\mu )\to P_{E1}(s,\mu \right) \end{array}$
It is supposed that primary emotions act as a protective response, i.e., as a safer mechanism that attempts to minimize risks by avoiding unknown situations (so, the probability of the action taken *E*_1_(*s*) = μ being correct, i.e., μ = μ*, will be low). **Definition:** The secondary emotional predictive ability, denoted by *P*_*E*2_, is represented as:
$\begin{array}{l} \;P_{E2}:\Omega \times E_{2}\to [0,1]\\ \left(s,E_{2}(s)=\mu )\to P_{E2}(s,\mu \right) \end{array}$
$P_{E_{2}}$ (*s*, μ) is the ability of taking right decisions based on acquired somatic markers. In most situations, the inferences on good choices are correct (i.e., the probability of the action taken, *E*_2_(*s*) = μ, being correct μ = μ*, is high) but, in other cases, wrong decisions occur as a consequence of rejecting suitable choices as well as failing to reject unsafe scenarios (i.e., the probability of the action taken, *P*_*E*2_(*s*) = μ, being correct, μ = μ*, is lower)

*Depending on the situation, only one of the mechanism is considered to be dominant at a given time*.

Let *X* be an agent, in a situation *s* ∈ Ω where the correct action is μ*. The agent is able to take two actions (μ_1_, μ_2_) but only the first one is correct (μ_1_ = μ*) and the second one is not.

If the agent were omniscient (i.e., it had the ability to know everything that can be known and the consequences of every decision made before choosing), then the probability of the action taken being correct would be *P_X_*(*s*, μ_1_) = 1, and by the same reasoning, the probability that the action taken is incorrect would be zero (*P_X_*(*s*, μ_2_) = 0).

However, in real life, in general, *P_X_*(*s*, μ_1_) < 1, and *P_X_*(*s*, μ_2_) > 0, when predicting the adequacy of the state achieved after performing a certain action (in some cases it can even happen that the agent estimates *P_X_*(*s*, μ_1_) < *P_X_*(*s*, μ_2_), i.e., considers the wrong option as the correct one). In real situations, therefore, only when the agent is certain that choosing the action “μ = μ*” is good, then *P_X_*(*s*, μ) = 1. Nevertheless, the “predictive ability” of an agent is lower.

For simplicity, it will be assumed that (i) there is no uncertainty about what the adequate actions are but that the uncertainty is restricted to the limitations of the agent’s cognitive abilities, and that (ii) convergence in probability is fulfilled, so the sample average converges almost surely to the expected value, i.e., *P_X_*(*s*, μ) = <*P_X_*(*s*, μ)> (note that this can be seen as a special case of the law of large numbers).

Consider a numerical example to clarify these notions:

• A reasoning mechanism that leaves the agent still unsure, say 20% of the time, is described as *P_R_*(*s*, μ = μ*) = 0.8.
• We assume that if the agent knows nothing about the current state (i.e., the situation is totally new), primary emotions act as an alarm mechanism by quickly deploying a protective response (i.e., conservatively making the agent perceive the situation as bad when uncertainty appears). In these conditions, the primary emotion’s ability to predict the correct action will be low (for instance, $P_{E_{1}}$(*s*, μ = μ*) = 0.2).
• If the agent knows nothing about the current situation but a correct somatic marker makes the agent interpret that a particular action could be correct, we can assume that the respective predictive ability will be good but, in general, not as high as the ability to predict by deliberation (e.g., $P_{E_{2}}$(*s*, μ = μ*) = 0.6).

### A formal framework to model dynamical dual systems

Decision-making in everyday life can be understood as a non-linear stochastic process: the effect of our actions and the risks involved in the world states are not always perfectly predictable. In general, it is considered that stochasticity derives from the shortcomings of the agent’s ability to predict the adequacy of the state achieved after performing a certain action. Furthermore, we also assume that actions and decisions can be modeled as discrete events in time. Taking these assumptions into consideration, a stochastic dynamical system is used to model the evolution of the set of states *s* ∈ Ω that a decision-making agent undergoes, as

$$s_{t+1}=f\left(s_{t},\xi_{t+1}\right),\;\text{with}\;\text{a}\;\text{random}\;\text{variable}\;\xi \left(t\right)\;\text{and}\;\forall t\in \mathbb{N}\;$$

Stochastic processes are characterized by a probability distribution Φ(*s*_*t*+1_, *t* + 1) describing the probability with which a possible state of the system, *s*_*t*+1_ ∈ Ω, can occur at time *t + 1*, given the previous sequence of states *s_t_*, *s*_*t*−1_, *s*_*t*−2_, …. Since we assume Markovian processes, the probability with which *s*_*t*+1_ ∈ Ω can occur at time *t* + 1, depends only on the state *s_t_* at the previous time *t*,

$$P\left(s_{t+1},t+1|s_{t},t\right),\;\;\forall t\in \mathbb{N}$$

A formal framework for the study of decision-making will be therefore described, in the most general way, in terms of the probability of a particular state *s_i_* ∈ Ω, in which the agent would be at time *t* + 1, as a function of where it was at the previous moment:

$$\Phi \left(s_{i},t+1\right)=\underset{j\neq i}{\sum }P\left(s_{i},t+1|s_{j},t\right)\cdot \Phi \left(s_{j},t\right),\forall s_{i},s_{j}\in \Omega ,\forall t\in \mathbb{N}\;$$

Note that we have defined the predictive capability of an agent, *P_X_*, as the result of a mechanism that enables it to make estimations of having a good/bad outcome following a particular action (see Cognitive Ability and Predictive Capability of an Agent in Appendix). In terms of this definition, the probability of transition in the Markovian process that describes the agent’s decision-making behavior, *P*(*s_i_*, *t* + 1 | *s_j_*, *t*) can be understood as equivalently to the “predictive ability of the agent” *P*(*s_t_*, μ), if the action μ: (i) is correct (μ = μ*) and (ii) allows the agent to move from *s_i_* to *s_j_*. If the probabilities of transition in the Markovian process are represented in matrix notation, then we have,

$$\Phi \left(t+1\right)=\Pi_{ij}\cdot \Phi \left(t\right)$$

where Π*_ij_* = *P*(*s_i_*, *t* + 1 | *s_j_*, *t*) does not depend on Ω but on the predictive ability of the agent (see the assumptions introduced in Cognitive Ability and Predictive Capability of an Agent in Appendix).

We can define a Markov chain by means of a linear and homogeneous system of differential equations. In general, any system that can be described by a set of p-state variables, *V* = (*v*_1_, …, *v_p_*), at *t* = 0 denoted as *V*_0_, that recursively generates a succession of other sets, *V*_1_, *V*_2_, …, *V_n_*, obtained by *V_n_* = *A*·*V*_*n*−1_, where *A* is a square matrix of order *p*, describing a linear and homogeneous system of differential equations.

It is said that a linear and homogeneous system of differential equations represents a Markov process, if the matrix coefficients of *A* are non-negative,

$$a_{ij}\geq 0,\forall i,j=1,2,\ldots ,p$$

and its columns add up to 1,

$$\overset{n}{\underset{i\;=\;1}{\sum }}a_{ij}\;=\;1,\forall j=1,2,\ldots ,p$$

In general, we can state that: (ii) knowing the behavior of a Markov process essentially means solving its associated differential equations systems, and (ii) every Markov process reaches a stationary regime (Rouché-Frobenius Theorem). We highlight that the last property will be essential to solve our model.

In dynamical terms, the mutual coupling between an agent and its environment modulates the agent’s behavior that results not only from internal processes but also from interaction. If we assume that a stationary state is reached, then the behavior of the agent can be described with respects to its internal processes as if it had an effective architecture (into which interactions with the environment and between processes have already been factored in). From this point of view, knowing the dynamics of the coupled system, describing the agent’s behavior properly or obtaining its “effective” architecture in the interaction is essentially the same.

Let us consider a simple example to clarify the method. We later discuss the results in general terms. We consider a case where the decision-making agent presents a dual architecture expressed by,

$$\left(\frac{2}{3}R⊕\frac{1}{3}E_{1}\right)$$

where we model the behavior of the agent with reasoning abilities and primary emotions and suppose, that – on average – a third of the decisions of the system are driven by *E*_1_ and the remaining two-thirds by *R* (see Figure 11).

From the moment in which the agent starts making decisions in a sequential manner, we can analyze the dynamics of its actions as a Markovian process. We start from a dual initial architecture (or equivalently, from the initial probability distribution of the Markov process) that can be expressed in a basis {*R*, *R*, *E*_1_} as,

$$V_{0}=\left(v_{R},v_{R},v_{E_{1}}\right)=\left(\frac{1}{3},\frac{1}{3},\frac{1}{3}\right)$$

In order to obtain the probability at time *t* = 1, denoted by *V*_1_, we can simply multiply the starting distribution by the matrix A,

$$V_{1}\;=\;A\cdot {\text{V}}_{0}$$

Since the system is Markovian, from a certain value *n*, it reaches a stationary regime,

$$V_{n+1}=A\cdot V_{n}$$

so the following will hold,

$$V_{n+1}=V_{n}$$

Therefore we can state that, after a transient period, the probability distribution of the Markovian process (or the effective architecture of the agent) will have reached a stable pattern, denoted by *V^st^*, in the coupling with the world. In our example, the Markovian matrix *A* that describes the systems is a three-square matrix,

$${\mathbb{P}}_{RE_{1}}=\left[\;\begin{array}{ccc} 0 & \left(1-P_{R}\right) & P_{R}\\ P_{E_{1}} & 0 & \left(1-P_{R}\right)\\ \left(1-P_{E_{1}}\right) & P_{R} & 0 \end{array}\right]$$

Solving the coupled system will consists in calculating the pattern that persists, i.e., the dynamic system equilibrium state. Then we look to obtain *V^st^*, the equilibrium distribution that becomes invariant under the action of ${\mathbb{P}}_{RE_{1}},$

$$V^{st}={\mathbb{P}}_{RE_{1}}\cdot V^{st}$$

Obtaining the *V^st^* consists in solving a typical eigenvalue problem with eigenvalue equal to 1,

$$\left({\mathbb{P}}_{RE_{1}}-I\right)\cdot V^{st}=0$$

that, in terms of our basis {*R*, *R*, *E*_1_}, it is expressed as,

$$V^{st}=\left(\;\begin{array}{c} v_{R}^{st}\\ v_{R}^{st}\\ v_{E_{1}}^{st} \end{array}\right)=\frac{1}{\eta \;}\left[\;\begin{array}{c} 1-P_{R}+P_{R}\cdot P_{E_{1}}\\ 1-P_{E_{1}}+P_{R}\cdot P_{E_{1}}\\ 1-P_{R}+P_{R}^{2} \end{array}\right]$$

where $\eta =3-P_{R}-2P_{E_{1}}+2P_{R}\cdot P_{E_{1}}+P_{E_{1}}^{2}\;\;$is a normalization constant so as to get $\Sigma_{i=1}^{3}\;v_{i}^{st}=1.$

In a simple numerical example where we take, for instance, for the prediction capability of the subsystems *R* and *E*_1_, the values $P_{E_{1}}=0.1$ and *P_R_* = 0.8 respectively, we obtain *V^st^* = {0.13, 0.47, 0.4} which reveals that the probability distribution in the equilibrium is not homogenous as the initial one, *V_0_* = {0.33, 0.33, 0.33}.

In other words, couplings between the emotional and the rational contribution to the cognitive architecture of the agent, forces the rational ability to decrease (in the example, from “weighing” 0.66 to 0.60) whereas the primary emotional part has a greater effect than its weight on the effective structure (the effect of primary emotions is greater than a 1/3). The components of this eigenvector can be understood as the new proportions between the subsystems 1 and 2 in the effective dual architecture of the agent.

Let us now consider that somatic markers are added to the initial system. We assume that there is, on average, a balance between the previous system and the new component $\left(\beta =\frac{1}{2}\right),$ so the global architecture of the agent can be represented by,

$$\left(\frac{1}{2}RE_{1}⊕\frac{1}{2}E_{2}\right)$$

We keep the notation for the previous case,

$${\mathbb{P}}_{RE_{1}}=\left[\;\begin{array}{ccc} 0 & 1-P_{R} & P_{R}\\ P_{E_{1}} & 0 & 1-P_{R}\\ 1-P_{E_{1}} & P_{R} & 0 \end{array}\right]$$

and use a similar notation for the Markov matrix that characterizes a pure somatic-marker process, such that

$${\mathbb{P}}_{E_{2}}=\left[\;\begin{array}{ccc} 0 & 1-P_{E_{2}} & P_{E_{2}}\\ P_{E_{2}} & 0 & 1-P_{E_{2}}\\ 1-P_{E_{2}} & P_{E_{2}} & 0 \end{array}\right]$$

the stable probability distribution in the combined Markov process is expressed as,

$$U^{st}={\mathbb{P}}_{RE_{1}E_{2}}\cdot U^{st}\;$$

where the new matrix will be,

$$\begin{array}{llll} & {\mathbb{P}}_{RE_{1}E_{2}}= & & \\ & \;\left[\;\begin{array}{ccc} 0 & 1-\frac{1}{2}\left(P_{R}+P_{E_{2}}\right) & \frac{1}{2}\left(P_{R}+P_{E_{2}}\right)\\ \frac{1}{2}\left(P_{E_{1}}+P_{E_{2}}\right) & 0 & 1-\frac{1}{2}\left(P_{R}+P_{E_{2}}\right)\\ 1-\frac{1}{2}\left(P_{E_{1}}+P_{E_{2}}\right) & \frac{1}{2}\left(P_{R}+P_{E_{2}}\right) & 0 \end{array}\right] & & \end{array}$$

and the stationary solution,

$$\begin{array}{llll} & U^{st}=\left(\;\begin{array}{c} u_{R}^{st}\\ u_{R}^{st}\\ u_{E_{1}}^{st} \end{array}\right)= & & \\ & \;\frac{1}{\nu \;}\left[\;\begin{array}{c} 1-\frac{1}{2}\left(P_{R}+P_{E_{2}}\right)+\frac{1}{4}\left(P_{R}+P_{E_{2}}\right)\cdot \left(P_{E_{1}}+P_{E_{2}}\right)\\ 1-\frac{1}{2}\left(P_{E_{1}}+P_{E_{2}}\right)+\frac{1}{4}\left(P_{R}+P_{E_{2}}\right)\cdot \left(P_{E_{1}}+P_{E_{2}}\right)\\ 1-\frac{1}{2}\left(P_{R}+P_{E_{2}}\right)+\frac{1}{4}{\left(P_{R}+P_{E_{2}}\right)}^{2} \end{array}\right] & & \end{array}$$

where $v=3-\frac{1}{2}\left(P_{R}+P_{E_{2}}\right)-\left(P_{R}+P_{E_{2}}\right)+\frac{1}{2}\left(P_{R}+P_{E_{2}}\right)\cdot \left(P_{E_{1}}+P_{E_{2}}\right)+\frac{1}{4}{\left(P_{R}+P_{E_{2}}\right)}^{2}$ is a normalization constant. We consider, as before, the agent’s initial architecture {0.33, 0.33, 0.33} in the basis {*R*, *R*, *E*_1_}.

If somatic-markers *E*_2_ are added with $\beta =\frac{1}{2}$ and we take, for example, $P_{E_{1}}=0.1,$ $P_{E_{2}}=0.6,$ and $P_{R}=0.8,$ we obtain that the effective architecture of the coupled somatic marker agent is given by $\left(u_{R}^{st},\;u_{R}^{st},\;u_{E_{1}}^{st}\right)=\left(0.25,\;0.40,\;0.35\right).$ Again, we can, see how the effect of emotions (in this case, secondary emotions) changes the structural contribution made for each subsystem in the effective architecture).

Let us now consider the previous *RE*_1_ agent in the one-dimensional situation referred in Figure 3. Although we represent the set of possible states for the agent as {…, *s*_*k*−1_, *s_k_*, *s*_*k*+1_, …} assuming that, on average 2/3 of the time, the agent will make a decision using the subsystem *R*, and 1/3 of time, the subsystem *E*_1_, we can reduce the analysis of the dynamics of a simpler process (a three-step transitions process),

$$\ldots s_{0}\overset{P_{01}}{\to }s_{1}\overset{P_{12}}{\to }s_{2}\overset{P_{20}}{\to }\ldots .$$

In order to obtain the probability of the agent making 3 transitions after 3 iterations we sum over all states in proportion to the probability of movement in the forward direction. If we express it in terms of the initial architecture of the agent, an intuitive interpretation can lead us to wrongly consider the summation performed should be arithmetic,

$$\Phi \left(\left\{s_{0}\overset{P_{01}}{\to }s_{1}\overset{P_{12}}{\to }s_{2}\overset{P_{20}}{\to }\right\},t=3\right)=v_{R}\cdot P_{R}+v_{R}\cdot P_{R}+v_{E_{1}}\cdot P_{E_{1}}$$

however, we must calculate,

$$\Phi \left(\left\{s_{0}\overset{P_{01}}{\to }s_{1}\overset{P_{12}}{\to }s_{2}\overset{P_{20}}{\to }\right\},t=3\right)=v_{R}^{st}\cdot P_{R}+v_{R}^{st}\cdot P_{R}+v_{E_{1}}^{st}\cdot P_{E_{1}}$$

where each component’s contribution is summed over the steady state probability vector $V^{st}=\left(\upsilon_{R}^{st},\upsilon_{R}^{st},\upsilon_{E_{1}}^{st}\right).$ If we suppose that choosing the right path implies going through states from left to right, then the expected value is also the definition of the predictive ability (see Table A2). Therefore, the result of calculating the probability of the agent going across the sequence $\left\{s_{0}\overset{P_{01}}{\to }s_{1}\overset{P_{12}}{\to }s_{2}\overset{P_{20}}{\to }\right\},$ assuming that this sequence is correct, is a measure of the predictive capability *P_X_* of the decision-making agent. The previous expression can be reformulated into,

$$P_{X}\left(\frac{2}{3}\cdot P_{R}⊕\frac{1}{3}\cdot P_{E1}\right)\neq \frac{2}{3}\cdot P_{X}\left(P_{R}\right)+\frac{1}{3}\cdot P_{X}\left(P_{E1}\right)$$

where, by definition, *P_X_*(*P_R_*) = *P_R_* and $P_{X}\left(P_{E_{1}}\right)=P_{E_{1}}$ (see Cognitive Ability and Predictive Capability of an Agent in Appendix). For instance, substituting the values *P_R_* = 0.8 and $P_{E_{1}}=0.1,$ and using the stationary distribution of probability, it is easy to conclude that the predictive ability of the agent will be *P_X_* = 0.52 and not $P_{X}=\left(0.66\cdot P_{R}+0.33\cdot P_{E_{1}}\right)=0.56.$

Consequently, we have shown the effect of how the predictability *P_X_* is affected by the interaction with the environment. The non-linear framework therefore shows that the global predictive ability of the agent is not a linear combination of the performance of each component (Harmer et al., 2002; Parrondo and Dinis, 2004). This can be explained intuitively: the negative effect of the emotional part traps the system and does not let the rational part spread over time as much as we could guess from looking at its representation (two thirds) in the system’s structure. Not being able to “move away” easily from states that demand an emotional cautionary response implies that the agent finds itself in those states and their immediately previous neighbors with greater frequency than expected from a uniform, independent sampling (what we describe as non-additive effects). Warning mechanisms can reduce the probability of using the predictive machinery more than we could establish by its proportional representation.

Thus, we could mistakenly think that, when considering a “somatic marker agent” (with α = 2/3 and β = 1/2, see Section 4 for details), the expected asymptotic value of the predictive ability is a linear combination of the predictive abilities from each component, however as the previous example, if we take $P_{E_{1}}=0.1$ and *P_R_* = 0.8 (it has been shown that for these values, $P_{RE_{1}}=0.52$) and add $P_{E_{2}}=0.6,$ the linear approach tells us that *P_X_* = 0.58, whereas in a non-linear framework, we obtain *P_X_* = 0.68.

From the effective architecture perspective linearity conditions are not verified as in the case of the isolated agent structure. In general, in coupled agents,

$$f\left(a\cdot A+b\cdot B\right)\neq a\cdot f\left(A\right)+b\cdot f\left(B\right)$$

In particular, we focus on the case in which the agent’s predictive abilities are defective, i.e., *P_X_* < 1/2 (i.e., the agent makes bad decisions most of the time).

Let us consider the somatic marker agent introduced previously, with predictive abilities $P_{RE_{1}}$ and $P_{E_{2}}.$ We are interested in exploring whether: (i) assembling defective components $\left(P_{E_{2}}<1∕2\right),$ (ii) to build the emotional-cognitive architecture of an agent ($P_{RE_{2}}\;<$ 1/2), (iii) can produce a reliable favorable behavior. We require additionally that $\left(P_{E_{2}}<P_{RE_{1}}\right),$ (where $P_{E_{2}}<1∕2$ means that we register experiences wrongly and $P_{E_{2}}<P_{RE_{1}}$ means that decisions made using secondary emotions lead us to make more mistakes than we do without somatic markers). This can be viewed as the mathematical version of Damasio’s main conclusion from empirical research with frontally damaged patients (Damasio, 1994, p. 221).

We prove that when then agent is coupled to the environment, in certain cases, wrong somatic markers can improve decision-making processes through the non-linear coupling of less than beneficial systems (Harmer et al., 2005). We present analytical results of the conditions under which this counterintuitive effect occurs for the particular set of parameters used in this paper (α = 2/3 and β = 1/2) that we are using for analytic convenience. Thus, for this effect to happen, the following inequalities need to be satisfied simultaneously:

- The first inequality represents a (reasoning + primary emotional) agent with low predictive capability
  $$V^{st}={\mathbb{P}}_{RE_{1}}\cdot V^{st}\;\Rightarrow \;P_{R}\cdot \left[v_{R}^{st}+v_{R}^{st}\right]+P_{E_{1}}\cdot \left[v_{E_{1}}^{st}\right]<1∕2$$
- The second inequality indicates that as wrong somatic markers are incorporated, the predictive ability is also worse than baseline
  $$W^{st}={\mathbb{P}}_{E_{2}}\cdot W^{st}\;\Rightarrow P_{E_{2}}\cdot \left[w_{R}^{st}+w_{R}^{st}+w_{E_{1}}^{st}\right]<1∕2$$
- The third inequality represents how the previous systems are combined to define the architecture of an agent with somatic markers. The inequality is reversed resulting in a better average performance
  $$\begin{array}{llll} U^{st} & ={\mathbb{P}}_{RE_{1}E_{2}}\cdot U^{st}\;\Rightarrow & & \\ & \;\frac{1}{2}\left(P_{R}+P_{E_{2}}\right)\cdot \left[u_{1}^{st}+u_{2}^{st}\right]+\frac{1}{2}\left(P_{E_{1}}+P_{E_{2}}\right)\cdot \left[u_{3}^{st}\right]>1∕2 & & \end{array}$$

Obtaining a solution requires finding probabilities that satisfy the three inequalities.

In order to easily solve the system of equations, it can be simplified as follows (a similar analysis can be consulted in Harmer et al., 2002; Parrondo and Dinis, 2004). Let us consider the stationary predictive ability of the dual deliberative-emotional agent, that is required that $P_{RE_{1}}<1∕2,$ or alternatively

$$P_{RE_{1}}<\left(1-P_{RE_{1}}\right)$$

In order for the second condition to be satisfied we would need $P_{E_{2}}<1∕2,$ and therefore,

$$P_{E_{2}}<\left(1-P_{E_{2}}\right)$$

Finally, it would be needed to satisfy the third inequality that,

$$P_{RE_{1}E_{2}}>\left(1-P_{RE_{1}E_{2}}\right)$$

Substituting the stationary distributions in these expressions, we have

$$\begin{array}{ccc} P_{E_{1}}\cdot P_{R}^{2} & < & \left(1-P_{E_{1}}\right)\cdot {\left(1-P_{R}\right)}^{2}\\ P_{E_{2}} & < & \left(1-P_{E_{2}}\right)\\ \begin{array}{c} 12\left(P_{E_{1}}\cdot P_{E_{2}}\right)\cdot \\ {\left[12\left(P_{R}+P_{E_{2}}\right)\right]}^{2} \end{array} & > & \begin{array}{l} \left[1-12\left(P_{E_{1}}+P_{E_{2}}\right)\right]\cdot \\ {\left[1-12\left(P_{R}+P_{E_{2}}\right)\right]}^{2} \end{array} \end{array}$$

Taking, for instance, *P_R_* = 0.9, *P_E_* = 0.01, and $P_{E_{2}}=0.45,$ it can be easily demonstrated that the previous three inequalities fulfill simultaneously. That is to say the incorporation of wrong secondary emotions (with predictive capability smaller than 1/2) can improve the predictability of the global system.
